# The definition and classification of pneumonia

**DOI:** 10.1186/s41479-016-0012-z

**Published:** 2016-08-22

**Authors:** Grant Mackenzie

**Affiliations:** 1 Basse Field Station, Medical Research Council Unit, The Gambia, c/o MRC Unit, PO Box 273, Banjul, Gambia; 2 Murdoch Childrens Research Institute, Melbourne, Australia; 3 London School of Hygiene and Tropical Medicine, Keppel St, London, UK

**Keywords:** Pneumonia, Definition, Classification

## Abstract

Following the publication of a volume of *Pneumonia* focused on diagnosis, the journal’s Editorial Board members debated the definition and classification of pneumonia and came to a consensus on the need to revise both of these. The problem with our current approach to the classification of pneumonia is twofold: (i) it results in widespread empirical, and often unnecessary, use of antimicrobials that contributes to pathogen resistance; and (ii) it contributes to heterogeneity among the groups of subjects compared in research, causing misclassification bias and mixtures of effects that threaten internal validity. After outlining the problem of classification, this commentary describes the strengths and weaknesses of a range of systems for the classification of pneumonia. The commentary then calls for debate to generate consensus classifications in the field, proposing a working definition and way forward focusing on the following three points: (i) pneumonia should be defined as an acute infection of the lung parenchyma by various pathogens, excluding the condition of bronchiolitis; (ii) defining pneumonia as a group of specific (co)infections with different characteristics is an ideal that currently has limited use, because the identification of aetiologic organisms in individuals is often not possible (however, the benefits of classifying pneumonia into specific, more homogenous phenotypes should be carefully considered when designing research studies); and (iii) investigation of more homogenous pneumonia groupings is achievable and is likely to yield more rapid advances in the field.

## Background

Volume 5 of *Pneumonia* was a theme issue on the diagnosis of pneumonia. Papers focused on the diagnostic roles of chest radiography [1], rapid analysis of biological samples [2], severity scores [3], and the electronic collection of multiple clinical input data with rapid algorithmic analysis [4]. An ensuing discussion between some of the *Pneumonia* Editorial Board members reflected on the lack of clarity around the definition and classification of pneumonia in the published papers and in the field of pneumonia research.

## Summary of opinions from the editorial board discussion

A number of members of the *Pneumonia* Editorial Board engaged in a discussion and were in agreement that the lack of an accepted and widely used definition or classification of pneumonia is a significant problem. However, there was less agreement on how pneumonia should be defined and classified and how this issue should be tackled. Perspectives from high-income settings included the poor correlation between radiologic appearance and systems for International Classification of Disease (ICD) for hospital admissions, and an appeal for a definition that recognises certain aspects particular to older adults. The diagnosis of pneumonia in the intensive care setting was noted as being particularly unreliable, although the evolving use of lung ultrasound offers more clarity. It was argued that the popular use of the term, ‘community-acquired pneumonia’ does little to advance our understanding of the condition. The medical literature includes the term ‘pneumonia’ or ‘pneumonitis’ in a collection of diagnostic terms for a number of conditions that are not related to infection or have an unknown cause, which illustrates the lack of consensus in the definition of pneumonia. The Editorial Board members who participated in the discussion would most likely agree that pneumonia is an acute infection distinguishable from chronic infections, although this should not be confused with several other acute lower respiratory tract infections with well-recognised and distinguishable patterns, such as bronchiolitis and bronchitis.

So, how can the field of pneumonia care and research overcome the current lack of clarity concerning definition and classification? One suggestion towards a definition of pneumonia was to take a descriptive approach: explaining anatomy and the respiratory ecosystem; describing what is meant by ‘pneumonia’; listing the causative organisms within the relevant context, and then proceeding to clinical definition(s). There was a call to reach a consensus on definitions of pneumonia in both resource-limited and well-resourced settings.

## Background

Pneumonia was first described by Hippocrates [5] (460–370 BC). The first descriptions of its clinical and pathological features were made 22 centuries later in 1819 by Laennec [6] while Rokitansky [7] in 1842 was the first to differentiate lobar and bronchopneumonia. During the next 47 years at least 28 terms were used to identify pneumonia [8], and by 1929 the total number of terms listed in the Manual of the International List of Causes of Death had grown to 94, with 12 sub-terms [9]. The ICD-10 classification of diseases has removed some of the historical descriptive terms and ‘pneumonia’ is listed as the primary term in seven codes (J12–18) but it is also a descriptive term in seven other codes relevant to specific infectious and non-infectious aetiologies, times of life and complications of diseases and procedures [10]. ICD-10 codes usually include subcategories so there are still many classifications for pneumonia. It is also of note that ‘other acute lower respiratory infections’ comprise three other codes (J20–22) for acute bronchitis, bronchiolitis and unspecified conditions.

## Characterisation of the problem and its magnitude

Harrison’s textbook of internal medicine defines pneumonia as an infection of the pulmonary parenchyma caused by various organisms. It states that pneumonia is not a single disease but a group of specific infections, each with a different epidemiology, pathogenesis, presentation and clinical course [11]. Harrison’s textbook describes the pathologic demarcation between lobar and bronchopneumonia but concludes that the classification of pneumonia is best based upon the causative microorganism. The textbook also describes that the specific microbial aetiology remains unknown in more than a third of patients, although it is common in children for a blood culture to be the only test performed to provide a specific diagnosis, which may only be positive in 5–10 % of patients and up to 20 % in the most severely ill patients [12].

The lack of accepted, widely understood and commonly used definition(s) for pneumonia causes a fundamental problem where related but heterogeneous pathologies and clinical phenotypes are poorly classified. The lack of clear classification results in difficulty with clinical decision making and a potential for poorly formulated research. The magnitude of this problem is most evident in the common inability to identify the infectious organism(s) causing lung infection, necessitating empiric antibiotic therapy. If a specific diagnosis could be made, specific therapy could be provided which would be of similar efficacy to empiric wide spectrum therapy [13] and avoid millions of prescriptions of broad-spectrum antibiotics and the associated risks of antibiotic resistance.

The magnitude of the problem is less evident in the field of pneumonia research. In a qualitative sense, the problem may be distilled to a lack of homogeneity in clinical and pathological phenotypes under investigation. In studies of heterogeneous groups the research problems that may arise include an inability to determine aetiology due to a limited range of methods; pathology or microbiology with disparate patterns; and conflicting results between studies that investigate risk factors, diagnostic methods or treatments. Heterogeneous groups may result in disparate and unfocused studies, which fail to target the most important types of pneumonia and the most important questions, and make limited contributions. In epidemiologic terms, investigation of heterogeneous groups will, to a lesser or greater extent, threaten the internal validity of studies. When heterogeneous groups are studied, invalid estimates of effect occur due to misclassification bias [14]. In the field of pneumonia research, determining aetiology is a common difficulty. For example, in the absence of specimens from the lung, studies of aetiology may misclassify causality to organisms detected in nasopharyngeal or sputum samples—in this situation, misclassification bias occurs due to the difficulty in accurately determining the aetiology of lung infection.

Epidemiologic studies are often designed to sample participants from a target population so that the study population is ‘representative’ of the target population. However, taken to an extreme, the pursuit of representativeness can defeat the goal of identifying causal biological relations. In laboratory science, it is routine for investigators to conduct experiments using animals with characteristics selected to enhance the validity of the experimental work rather than to represent a target population. Concerns about generalisability only become important after it is accepted that the study results are valid for the restricted group. Likewise, epidemiologic study designs are stronger if participant selection is guided by the need to make a valid comparison, which may call for severe restriction of eligibility to a narrow range of characteristics, rather than attempting to make the participants generally representative [14]. Statistically speaking, selection of participants who are representative of larger populations will often make it more difficult for internally valid inferences to be made, due to misclassification/heterogeneity of cases and an inability to control for confounding by factors that vary within those populations.

## Minimising problematic heterogeneity—definitions and classification of pneumonia

To minimise the threats to the validity of research outlined above, homogeneous study groups should be selected with respect to clinical phenotype, pathology and important confounders. Once effect estimates are established by studies designed to maximise validity, generalisation to other groups becomes simpler. To a large extent, generalisation is a question of whether the factors that distinguish populations from the study group somehow modify the effect in question. To answer this question, epidemiologic data will be of help, but other sources of information such as pathophysiology may also play an important role. In relation to pneumonia research, the internal validity of studies will be improved if participants are more homogenous with respect to factors associated with the outcome. For example, when comparing antibacterial therapies in patients with pneumonia, the internal validity of the study will be improved if the patient group is restricted to those with a proven bacterial cause; antibacterial therapy cannot benefit patients with viral pneumonia. The question of generalising study results to wider populations is a separate consideration. Heterogeneity of research participants is not always bad and may be required when findings need to be generalised to wider populations. The focus here is to illustrate the value of studying homogenous groups when the current lack of clarity in the classification of pneumonia results in a tendency towards the inclusion of heterogenous groups.

Two examples of pneumonia research that have substantially advanced the field illustrate the scientific benefit of using restrictive criteria to study homogenous patient subgroups. Firstly, between 1971 and 1981 a collection of childhood pneumonia aetiology studies using lung aspiration established, without a doubt, that the aetiology in cases of substantial lobar pneumonia (an homogenous subgroup) was bacterial in 30–80 % of cases and pneumococcal in 10–50 % [15]. A more recent study by McNally and colleagues [12] extensively investigated children with severe or very severe pneumonia with half having failed therapy (a homogenous subgroup), and found that 18 % had more than one aetiologic bacterial organism, thus establishing the paradigm of co-infection in pneumonia.

To attain a homogeneous group of participants, research studies must classify patients according to some given criteria. Table 1 describes several systems for the classification of pneumonia and also notes their advantages and disadvantages. It is clear that all of the systems of classification have significant deficiencies, primarily relating to an inability to determine the aetiology of cases of pneumonia, and substantial heterogeneity of aetiology, phenotype and pathology. A theme also emerges where the classification systems that are designed to guide clinical care and treatment (e.g. the World Health Organization [WHO], National Institutes of Health [NIH], and Harrison’s), are the most prone to heterogeneity. Given that empiric prescription of antibiotics and antimicrobial resistance are such a concern and that pneumonia research using definitions and classifications that lead to substantial heterogeneity is relatively common, it would appear that a fresh perspective would benefit the field.

**Table 1:** Methods of pneumonia classification and their advantages and disadvantages

Classification	Description of classification	Advantages	Disadvantages
WHO [16]	Pneumonia: Age 2–59 months with cough or difficult breathing and fast breathing and/or chest in-drawing. Severe pneumonia: pneumonia with any danger sign	*Clinical:* simple programmatic implementation to guide treatment *Research:* easy to enrol patients and findings directly generalisable	*Clinical:* no definition of aetiology, high levels of empiric antibiotic therapy *Research:* highly heterogeneous including viral, bacterial and other aetiologies
NIH [17]	Community/hospital-acquired, health care-associated, aspiration, and atypical (caused by *Legionella, Mycoplasma, Chlamydia*)	*Clinical:* simple, guides empiric therapy *Research:* easy to enrol patients, findings directly generalisable	*Clinical:* little definition of aetiology or pathology, empiric antibiotic therapy *Research:* heterogeneous phenotypes
Pathology	Acute inflammation of lung parenchyma, inflammatory alveolar infiltrate	*Clinical:* resolve cases of difficult diagnosis *Research:* highly homogenous	*Clinical:* limited availability and relevance *Research:* difficult to enrol patients
ICD-10	Uses clinical and laboratory diagnoses with known or unknown aetiology and many potential classifications	*Clinical:* not used clinically, primarily used for audit and administration *Research:* Analyses of clinical databases	*Clinical:* limited relevance *Research:* little definition of aetiology, heterogeneous, not systematic
Harrison’s textbook [11]	Infection of pulmonary parenchyma by various pathogens, not a single disease. Terms lobar or bronchopneumonia not recommended. Clinical categories: community-acquired, nosocomial, aspiration	*Clinical:* encourages aetiologic diagnosis and guides empiric therapy *Research:* aetiologic diagnosis provides homogeneity, findings are directly generalisable	*Clinical:* difficult to confirm aetiology, substantial empiric antibiotic therapy *Research:* difficult to enrol patients with a single aetiology, clinical categories give heterogeneous aetiology and phenotype
Clinical	Features: Age, acute/chronic, bronchiolitis, nosocomial, recurrent, comorbidity, HIV-related, complications, severity, mortality	*Clinical:* multiple inputs to guide treatment *Research:* may be easy to enrol patients, flexible, may define ‘important’ subgroups	*Clinical:* no aetiology, empiric therapy *Research:* heterogeneity, not standardised, difficult to generalise
Chest radiograph	Interstitial/alveolar/lobar/air bronchogram WHO: dense, fluffy consolidation of entire lung or portion of a lobe; often with air bronchograms and possibly pleural effusion [15]	*Clinical:* supports viral or bacterial aetiology, identifies complications *Research:* some homogeneity and alignment with aetiology, standardised	*Clinical:* availability, time, expense *Research:* some difficulty enrolling patients, heterogeneous aetiology, unable to detect co-infection
Ultrasound	Subpleural consolidation, B-lines, pleural line abnormalities, pleural effusion, air bronchogram	*Clinical:* fast, no radiation, for complications *Research:* simpler than radiograph, some homogeneity	*Clinical:* availability, no aetiology *Research:* detection in non-peripheral lung, not standardised, heterogeneity
Microbiology	Culture of blood, lung/pleural aspiration, BAL Bacterial – viral – co-infection	*Clinical:* directs specific therapy *Research:* homogenous	*Clinical:* slow, limited detection *Research:* difficult to enrol patients
Serology/antigen	Blood, urine, NPS (*Legionella, S. pneumoniae*)	Rapid, pathogen-specific	Range/sensitivity of tests, misclassification
CRP	High CRP correlates with bacterial aetiology	Increased sensitivity for bacterial disease	Optimal threshold unclear, no aetiology

*BAL* Broncho-alveolar lavage, *CRP* C-reactive protein, *NPS* Nasopharyngeal sample

## A way forward

In the current setting of limited diagnostic methods, this commentary proposes three points to ameliorate the difficulties with the definition and classification of pneumonia. A working definition and approach to classification is proposed to guide future research and, to a lesser extent, clinical care. Each of the three points includes a qualifying statement that, if heeded, should benefit the field of research.

### One

Pneumonia should be defined as an acute infection of the lung parenchyma by one or co-infecting pathogens, but excluding the well-defined condition of bronchiolitis, the primary cause of which is almost always a viral agent.

### Two

It should be accepted that defining pneumonia as a group of specific (co)infections with different characteristics is an ideal, but this ideal currently has limited use because the identification of aetiologic organisms in individuals is often not possible. This statement is qualified, however, in that the classification of pneumonia into specific phenotypes using current or potential methods should be carefully considered when designing research studies. The study of more homogenous phenotypes is likely to provide better evidence for clinical care and more clear inference in research. Research should continue into the aetiologic diagnosis of pneumonia, as better understanding of aetiology and pathogenesis will improve our ability to prevent pneumonia and provide specific therapy.

An implication of point two is that research should define questions that can be answered in a valid manner. The inclusion of more homogenous phenotypes will minimise confounding and bias. Emphasis on inclusion of patient groups that are representative of wide populations may not advance the field as wished. It may be that a greater focus on studying high-risk groups, specific aetiologies, very severe cases, patients with consolidation, narrow age groups, etc., may provide a greater yield of knowledge to support targeted interventions. Thus, clinicians and public health researchers should consider how best to function in the arena of pneumonia treatment guidelines and policy as well as that of the biology, pathology, therapy and prevention of pneumonia in subgroups and subtypes of pneumonia. It needs to be considered how these two domains—public health policy for the treatment and prevention of pneumonia, and research to answer specific questions of pathogenesis, diagnosis, treatment and prevention—can better interact.

### Three

Classifications of pneumonia in clinical care and research will be limited by the means available but can be made more specific using the approaches described in Table 1, and combinations thereof (Table 1 is not a complete list of all available approaches). This statement is qualified by the knowledge that vaccine probe studies are potentially powerful instruments for the investigation of pneumonia aetiology and pathogenesis and investigators should take the opportunity to conduct such studies. Vaccine probe studies allow classifications of pneumonia which are impossible by any other means. A classic vaccine probe study was able to define the entity of ‘viral-associated pneumococcal pneumonia’ providing a substantial advance in our understanding of pneumonia pathogenesis and aetiology [18].

In summary, refining the definition and classification of pneumonia is a formidable task as multiple terms are used in multiple fields of medical practice and research. The dangers of poor classification of pneumonia are widespread empiric antibiotic therapy and heterogeneous groups in research, which have a tendency to influence the construction of research questions and studies. As a result, these research questions and studies may not provide clear answers. The aim of this commentary is to stimulate debate towards consensus classifications for clinical terminology, separating bronchiolitis from pneumonia, examining the value of the community- and hospital-acquired classification, and purposeful refinement of classifications based on microbiology, aetiology, radiology, severity, complications, important age groups and subgroups. In the interim, better ways to determine the aetiology of pneumonia need to be sought, and researchers should consider the benefits of using methods of classification to provide more homogeneous groups, the study of which is likely to provide clearer answers to research questions.
